# Regulation of circulating endocannabinoids associated with cancer and metastases in mice and humans

**DOI:** 10.18632/oncoscience.33

**Published:** 2014-04-30

**Authors:** Sebastian Sailler, Katja Schmitz, Elke Jäger, Nerea Ferreiros, Sabine Wicker, Katja Zschiebsch, Geethanjali Pickert, Gerd Geisslinger, Carmen Walter, Irmgard Tegeder, Jörn Lötsch

**Affiliations:** ^1^ pharmazentrum frankfurt/ZAFES, Institute of Clinical Pharmacology, Goethe-University Hospital, Frankfurt am Main, Germany; ^2^ Department of Hematology and Oncology, Krankenhaus Nordwest, Frankfurt am Main, Germany; ^3^ Occupational Health Service, University Hospital Frankfurt, Frankfurt am Main, Germany.

**Keywords:** Cannabinoids, Human, Mice, Metastasis

## Abstract

**Background and aims:**

Endocannabinoids may modify cancer development, progression and associated pain. We determined whether cancer-evoked dysregulations in this system become manifest in altered tissue and plasma endocannabinoids.

**Methods:**

Endocannabinoid changes due to cancer were explored in a local and metastatic syngeneic mouse melanoma model. Endocannabinoid stratification in human cancer was cross-sectionally assessed in the plasma of 304 patients (147 men, 157 women, aged 32 - 87 years) suffering from several types of cancer at Roman Numeral Staging between I and IVc, mostly IV (n = 220), and compared with endocannabinoids of healthy controls.

**Results:**

In mice with local tumor growth, ethanolamide endocannabinoids, i.e., anandamide (AEA), palmitoylethanolamide (PEA) and oleoylethanolamide (OEA) were downregulated, whereas 2-arachidonoylglycerol (2-AG) was increased. Upon spreading of the cancer cells particularly 2-AG steadily increased in parallel to disease progression while OEA modulated cell migration. Results translated into humans, in whom cancer was associated with a decreased AEA, increased 2-AG and increased OEA correlating with the number of metastases.

**Conclusions:**

The endocannabinoid system was subject to cancer-associated regulations to an extent that led to measurable changes in circulating endocannabinoid levels, emphasizing the importance of the endocannabinoid system in the pathophysiology of cancer.

## INTRODUCTION

**Figure 1:**
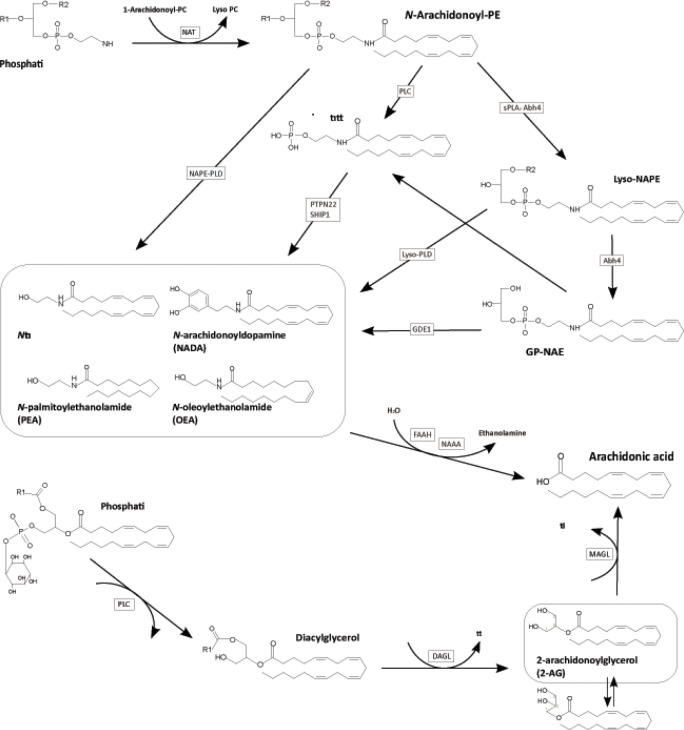
Pathways for the synthesis and degradation of N-actetylethanolamines (NAE) and 2-arachidonoylglycerol (2-AG) The first reaction of the NAE-synthesis is the N-acylation of phosphatidylethanolamine (PE), which is catalyzed by N-acyltransferase (NAT). The most important second step is a phospholipase (PDL)-type hydrolysis of the resultant N-acylethanolamine-phosphatidylethanolamine (NAPE-PE) by the NAPE-PLD leading to the formation of the NAEs, N-arachidonoylethanolamide, N-palmitoylethanolamide, and N-oleoylethanolamide and a related substance, N-arachidonoyldopamine (NADA). Other suggested pathways are a two-step process involving a PLC-like hydrolysis of N-arachidonoyl-PE with subsequent dephosphorylation of anandamide phosphate by PTPN22 and the double-deacylation of NAPE by α/β-hydrolase 4 (Abh4) with subsequent hydrolysis of the phosphodiester bond of the resultant glycero-phospho-NAE (GP-NAE) by glycerophosphodiester phosphodiesterase 1 (Gde1). The major pathway for the biosynthesis of 2-AG involves sequential hydrolysis of arachidonic acid-containing inositol phospholipids by PLC and diacylglycerol lipase (DAGL). The degradation of the NAEs as well as 2-AG is catalyzed by the N-acylethanolamine-hydrolyzing amidase (NAAA), fatty acid amide hydrolase (FAAH) and monoacylglycerol lipase (MAGL), respectively, and results in arachidonic acid.

Endocannabinoids (Figure 1) are involved in several ways in cancer pathophysiology including inflammation, tumor progression and pain. The lipid mediators arachidonoyl ethanolamide (anandamide, AEA) and 2-arachidonoyl glycerol (2-AG) exert their effects via agonist binding at cannabinoid CB_1_ and CB_2_ receptors [1] whereas structural relatives, e.g., palmitoylethanolamide (PEA) and oleoylethanolamide (OEA), exert their effects via orphan G-protein coupled receptors (e.g. GPR18, 55, 92 and 119 [2]) or nuclear receptors PPARγ and α [3].

Endocannabinoids inhibit tumor proliferation via direct effects on tumor cells [4] in breast, brain, skin, thyroid, prostate and colorectal cancers [5, 6] and via the suppression of angiogenesis and tumor invasion [7]. They contribute to failures of the immune system to attack tumors by favoring CB_2_ mediated polarization of tumor-associated macrophages and dendritic cells toward tumor tolerance [8, 9]. Particularly anandamide causes a silencing of macrophage-like cells and release of inhibitory cytokines [10, 11] whereas 2-AG acts oppositely [12]. Endocannabinoid release from tumors modifies the tumor environment, immune cell infiltration and tumor attack [13] and may favor tumor growth whereas high endocannabinoid concentrations may inhibit tumor growth [14-18].

Thus, several lines of evidence support that tumor development and progression may be associated with disturbances of the endocannabinoid balance. Endocannabinoids originating from tumors apparently contribute to the plasma endocannabinoid pool [19], however, without a consistent pattern [5, 6]. The present study addressed tumor associated regulations of endocannabinoids in mouse melanoma models and cancer cell migration assays. The observations were translated to humans by assessing cancer associated changes in circulating endocannabinoids in patients with several types and stages of cancer.

## RESULTS

### Endocannabinoid regulation associated with localized malignancies

**Figure 2:**
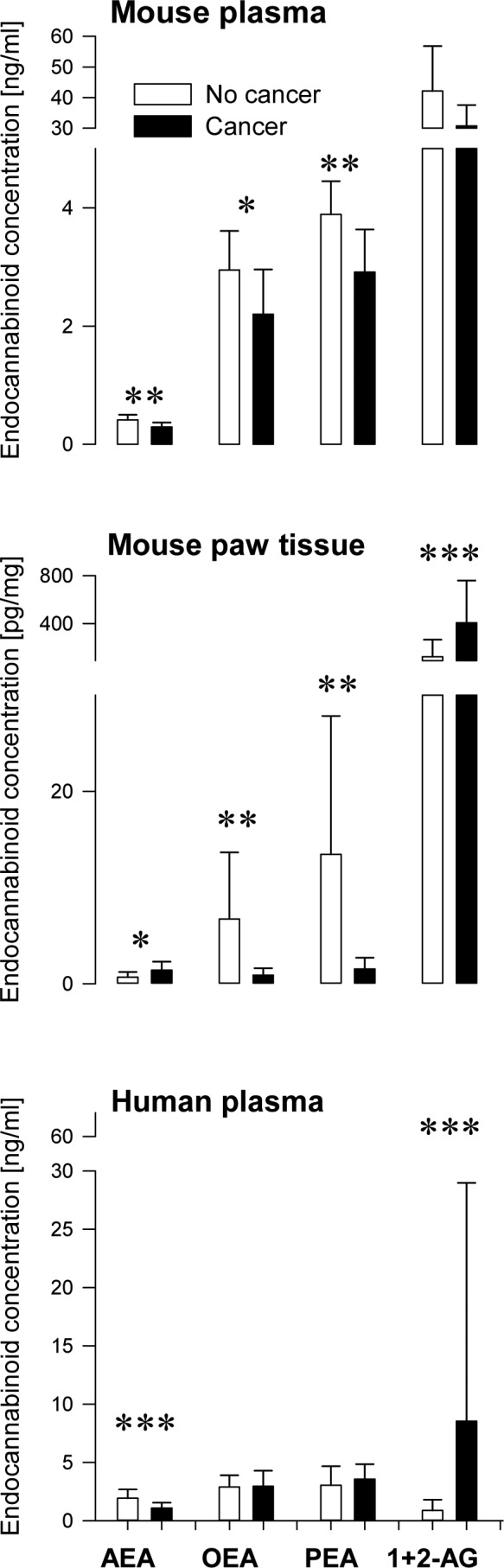
Plasma and tissue concentrations (means ± SD) of endocannabinoids in plasma of mice (top) and humans (bottom) and in the tumor environment of a local paw tumor in mice (middle) B16 mouse melanoma cells were injected into the left paw fat pad tissue. Plasma and paw tissue samples were obtained 14 days after tumor cell injection. In control mice, cell suspension medium without tumor cells was injected. Dissected tissue samples contained the tumor and immediately surrounding tissue. n = 8 - 9 mice for plasma, n = 18 mice for tissue. Human data was assessed cross-sectionally in aged and sex matched subsamples of patients (n = 44) and controls (n = 42)). Asterisks indicate significant group differences between (t-tests: *: p < 0.05, **: p < 0.01, ***: p < 0.001).

Plasma concentrations (Figure 2 top) of ethanolamide endocannabinoids (AEA, OEA and PEA) in mice with a local paw tumor were significantly lower as compared to control mice (t-tests: p < 0.009 for AEA, p = 0.046 for OEA and p = 0.007 for PEA). This was paralleled by the local concentrations in cancerous paw tissue (OEA: p = 0.003, PEA: p = 0.002 compared with the contralateral paw, Figure 2 middle). In contrast, plasma 2-AG concentrations were increased. While this tendency missed statistical significance (p = 0.063), it was significant with respect to local concentrations, i.e., at the tumor site the concentrations of 2-AG were significantly higher than at the control side (p < 0.001).

Endocannabinoid plasma concentrations could be obtained from 298 cancer patients. In age and sex matched subsets of subjects encompassing 42 controls and 44 cancer patients aged 45.3 ± 5.5 and 46.2 ± 5.6, respectively (t-test for age: p = 0.44; equal distribution of sexes; χ^2^ − test: p = 0.23), comparatively lower concentrations of AEA in patients (1.1 ± 0.6 ng/ml) than in the controls (1.9 ± 0.8 ng/ml) were identifiable (Mann-Whitney U-test: p < 0.001), similarly to the findings in mice. In addition, higher concentrations of 2-AG as seen in mice were also found in this sub-sample of cancer patients (patients: 8.5 ± 20.4 ng/ml, controls: 0.9 ± 0.9 ng/ml, U-test: p < 0.001; Figure 2).

### Endocannabinoid regulation associated with metastatic cancer

**Figure 3:**
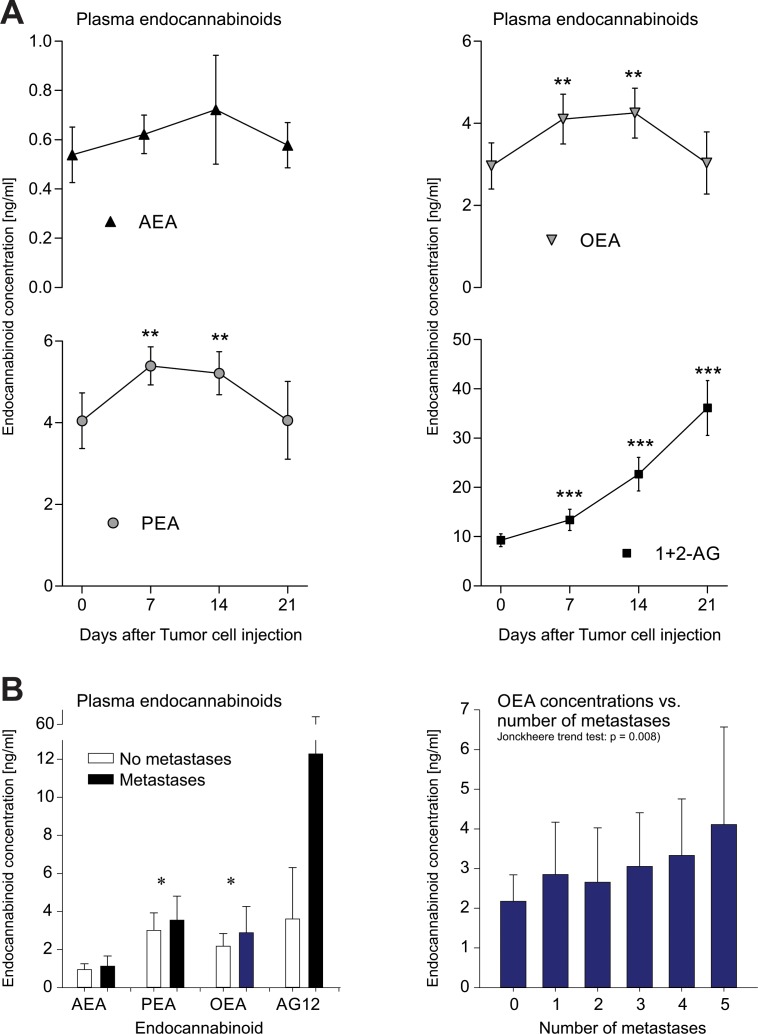
Changes in endocannabinoid plasma concentrations associated with metastases A: Plasma concentrations (mean ± SD) of endocannabinoids in metastatic mouse B16 mouse melanoma. Cells were injected via the tail vein and metastases were identified on the basis of the melanin production. They developed in lung, spleen, peritoneum and lymph nodes. Plasma samples were taken at the indicated times before and after tumor cell injection. B: Plasma endocannabinoid concentrations in cancer patients. Increased ethanolamide endocannabinoid plasma concentrations in the presence of metastases resembled alterations in mice (left). Asterisks indicate significant group differences between groups (t-tests after significant ANOVA: *: p < 0.05, **: p < 0.01, ***: p < 0.001 Bonferroni α-corrected (with exception of PEA)). Moreover, the concentrations of OEA (and PEA) increased significantly with increasing number of metastases (Jonckheere– Terpstra trend test: PEA: p = 0.005, OEA: p = 0.008).

**Figure 4:**
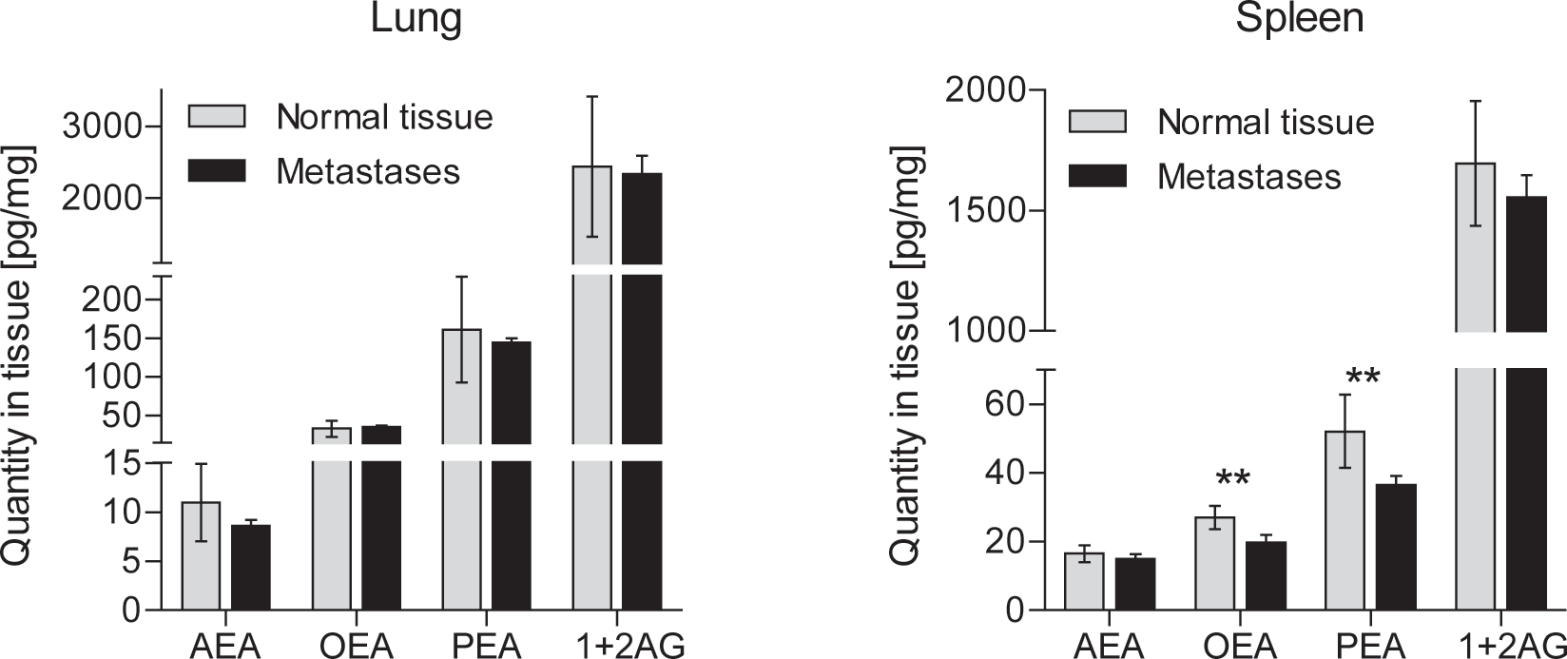
Tissue concentrations (mean ± SD) of endocannabinoids in metastatic mouse B16 mouse melanoma Cells were injected via the tail vein and metastases were identified on the basis of the melanin production. They developed in lung, spleen and lymph nodes. Metastases and corresponding control tissue samples were dissected at 4 weeks (n = 10 mice for plasma, 5-8 for tissue). Asterisks indicate significant differences versus baseline (plasma) or metastasis versus non-cancerous corresponding tissues (t-tests after significant ANOVA, p < 0.05).

**Figure 5:**
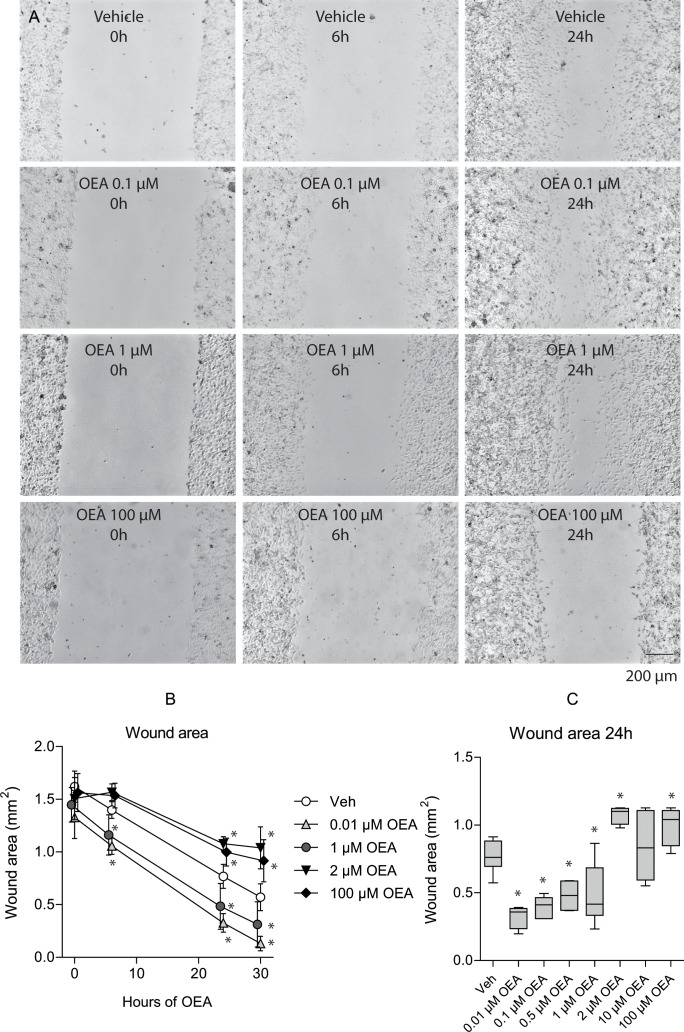
Effects of OEA on B16 cell migration and wound closure in culture Sub-confluent B16 cultures were scratched with a pipet tip causing ca. 1 mm wide wounds. Images were captured directly after scratching (0 h) and at 6, 24 and 30 h. The wound area was analyzed. A: Examples of the culture wound in vehicle and OEA treated cultures. B: Quantitative analysis of the time course of the wound area (mean ± SD). C: Box plots of the wound areas of vehicle and OEA treated cultures at 24h. The boxes show the 25^th^-75^th^ quantiles and the whiskers the 10^th^ - 90^th^. The line is the median. Asterisks indicate statistically significant differences versus vehicle treated cultures, p < 0.05. For comparison, human plasma concentrations were in the range of 5 - 20 nM, whereas paw concentrations in control animals ranged from 10 - 50 pmol/mg (~10 - 50 μM).

In the metastatic cancer mouse model, the plasma endocannabinoid concentrations initially increased (Figure 3 A; rm-ANOVA effect of the within-subjects factor “time”: AEA p = 0.038, OEA p = 0.000008, PEA p = 0.000002, 2-AG p < 0.000001; significant differences to baseline depicted in Figure 3). Primary sites of metastases were lung, spleen (Figure 4), peritoneum and lymph nodes. All mice developed metastases at multiple sites. Body weight remained constant up to 3 weeks after tumor cell injection. In the culture wound-healing assay on metastatic cancer OEA exerted dual concentration-dependent effects on tumor cell migration, with most OEA concentrations significantly differing from vehicle (Figure 5). Specifically, low concentrations (0.01 - 1 μM) promoted tumor cell migration and wound closure whereas high concentrations (2 - 100 μM) inhibited migration (wound area: rm-ANOVA factor “time”: df = 3,129, F = 601, p < 0.0001, factor “OEA concentration”: df = 7,120, F = 15.4, p < 0.0001, interaction “time” by “OEA concentration”: df = 21,120, F = 4.99, p < 0.0001). At 24 h, effects of OEA on the wound area differed depending on its concentrations (df = 7,42, F = 16.8, p < 0.0001; for further statistical details, see Figure 5).

Most of the 298 cancer patients had metastatic cancer (n = 273 versus n = 25 without metastases) and the number of metastases at different locations (Table 1) differed significantly between cancer types (configural frequency analysis: χ^2^ = 127, p < 10^−6^). The presence of metastases was associated with significantly higher concentrations of ethanolamide endocannabinoids (Figure 3B), in particular of OEA (t - test: p < 0.001 α-corrected) whereas the similarly directed difference in PEA was only significant at the uncorrected α-level (p = 0.047) and that of AEA was not significant (p = 0.111). Moreover, the concentrations of PEA and OEA were significantly correlated with the number of metastases (PEA: ρ = 0.165, p = 0.004, OEA: ρ = 0.155, p = 0.008). Their concentrations increased with increasing number of metastases (Jonckheere–Terpstra trend test: PEA: p = 0.005, OEA: p = 0.008). In contrast to the ethanolamide endocannabinoids, concentrations of 1+2-AG were not influenced by the number of metastases (p = 0.4).

**Table 1:** Patients' demographics, the actual Roman Numeral Staging and the number of metastases, grouped per cancer diagnosis

		Urologie / Prostatic cancer	Breast cancer	Lung cancer	Gastroin-testinal tumors	Hematological malignancies	Pancreatic cancer	Gynecological cancer	Skin cancer	Other malignancies	All	Statistical comparisons
**N**		42	57	48	79	18	30	15	6	9	304
**Men/ women [n]**		39/3	1/56	28/20	46/33	9/9	15/15	0/15	4/2	5/4	147 / 157	0.00015^a^
**Age [yrs]**		67.5 ± 11.9	62.2 ±9.3	63.9 ± 10.3	64.5 ± 10.7	64.8 ± 15.6	66.3 ± 7.3	66.9 ±6.2	53 ± 13.6	61.9 ±9.9	64.4 ± 10.6	0.049^b^
**Weight [kg]**		79.5 ± 15	70.3 ± 15	73.6 ± 17.1	72.2 ± 15.4	78.2 ± 17.5	66.6 ± 13.6	70.5 ± 15.7	77.5 ± 14.4	72 ±17.2	72.9 ± 15.8	0.03^b^
**BMI**		25.6 ±4.8	26.4 ±5.1	25 ±4.6	24.5 ±4.2	26.5 ±5.1	23 ±4.1	26 ±5.5	26.8 ± 5.6	24 ±5.1	25.2 ±4.7	0.053^b^
**Former/actual smokers [n]**		5/4	3/8	8/22	8/8	4/0	7/6	1/1	0/1	1/0	37/50	0.000007^c^
**Time since cancer diagnosis [month]**		61.7 ± 44	106.5 ± 84.1	17.5 ± 20.4	43.5 ±44.1	34.6 ± 36.4	16.9 ± 25	55.3 ± 34.5	48.5 ±45.8	66 ±92.7	51.9 ±59.3	<0.00001^b^
**Karnofsky status**		75.2 ±16.3	74.6 ±16	76.3 ± 15.2	77.2 ±14.8	79.4 ±18.3	78.3 ±15.6	81.3 ± 14.1	90 ± 16.7	73.3 ± 21.8	76.9 ± 15.8	0.414^b^
**Actual cancer stage**	I	2	1	0	0	0	0	0	0	0	3	-
	Ia	0	0	2	0	0	0	1	0	1	4	
	Ib	0	0	0	0	0	1	0	0	0	1	
	II	1	0	1	0	0	0	0	0	0	2	
	IIa	0	2	1	0	1	4	0	0	1	9	
	IIb	0	3	4	0	0	5	0	0	0	12	
	III	1	0	0	0	7	2	0	0	0	10	
	IIIa	0	1	1	10	0	0	1	0	0	13	
	IIIb	1	0	8	4	0	0	1	1	0	15	
	IV	37	50	31	57	4	17	12	5	7	220	
	IVa	0	0	0	4	0	0	0	0	0	4	
	IVc	0	0	0	3	0	0	0	0	0	3	
	n.a.	0	0	0	1	6	1	0	0	0	8	
**Metastases**	Liver	9	22	10	43	2	13	6	0	5	110	< 0.000001^d^
	Bone	23	34	9	7	9	0	1	0	2	85	
	CNS	2	6	6	2	0	0	0	2	0	18	
	Lung	13	17	20	29	1	3	2	2	5	92	
	Lymphatic	21	39	40	64	6	21	11	5	3	210	
	Other	7	14	14	23	2	8	10	2	2	82	
	All	75	132	99	168	20	45	30	11	17	597	

a Significances are given according to χ^2^ statistical comparisons among cancer types.

b In the case of ANOVA, the main effect of the factor "cancer diagnosis" is given.

c The numbers of never smokers, former smokers, and actual smokers were compared among cancers.

d results of a configural frequency analysis.

## DISCUSSION

Endocannabinoids are involved in the regulation of several physiological systems that are involved in tumor development and tumor growth [6] as demonstrated by a disturbance of endocannabinoids in cancer in general, and specifically in metastatic cancer [4-7]. We showed a downregulation of ethanolamide endocannabinoids following local cancer induction in mice translated into cancer patients. This decrease and the reciprocally directed increase in 2-AG might be interpreted as reflecting a tumor-evoked alteration of the microenvironment that possibly favors tumor growth. In mice plasma ethanolamide endocannabinoids re-raised during spreading of the cancer cells and 2-AG kept increasing. The increase was evoked in mice by intravenous injection of the tumor cells directly initiating metastatic disease. The role of metastases translated into differences in circulating ethanolamide endocannabinoids in patients, in whom endocannabinoids increased with the number of metastases.

Since plasma concentrations of endocannabinoids may point at an intact communication between brain, immune system, hormones, fat and muscle tissue [19], the present investigation suggested that this balance is deranged in cancer to a degree measurable in plasma despite many influences contributing to circulating endocannabinoids. Those include their production in peripheral tissues and the brain and their degradation depending on the functioning of several enzymes such as the fatty acid amide hydrolase (FAAH), the monoacylglycerol lipase (MAGL), the palmitoylethanolamide-preferring acid amidase and cyclooxygenases [23], lipoxygenases and cytochrome P450 enzymes [24]. It is conceivable that the metabolism of ethanolamide endocannabinoids was increased in cancer. For example, FAAH is known to be upregulated in prostate, pancreatic, colon and breast cancer [25-28]. In addition, COX-2 is also known to be upregulated in various types of cancer [29]. As some COX-2 inhibitors reduce the risk for colon cancer, which cannot entirely be explained by inhibition of prostaglandin synthesis [30], an effect mediated via the endocannabinoid system appears possible and would fit to the present observations, i.e., a reduction of oxidative degradation of ethanolamide endocannabinoids might contribute to the cancer protective effects of COX-2 inhibition. Similarly, substances that reduce the activity of FAAH such as flurbiprofen [31] and thus increase endocannabinoid concentrations exert anti-proliferative actions [32, 33]. In contrast to ethanolamide endocannabinoids, 2-AG may promote tumor invasiveness. It was suggested that this occurs by rapid metabolism to 12-HETE by 12/15 lipoxygenases, which, at least in prostate cancer, promotes tumor growth [34]. In addition, MAGL hydrolyzes 2-arachidonoylglycerol to generate an arachidonate precursor pool for prostaglandins [35], which also promote certain types of cancer [36].

Decreases of circulating endocannabinoid concentrations in patients with metastatic cancer were less pronounced than in patients with non-metastatic cancer. The sources of these endocannabinoids may be the spreading tumor cells themselves. Alternatively, activated immune cells may release the endocannabinoids when they are stimulated by circulating viable and dying tumor cells [37]. The observed time course of plasma endocannabinoid concentrations in the metastatic mouse model suggests an initial immune activation by large numbers of circulating tumor cells, which was followed by a state of anergy or immune cell redistribution as soon as the metastases were established. In the cross-sectional human metastatic cancer data, the association of OEA and PEA concentrations with the number of metastases hints at similar mechanisms as in mice. An involvement of endocannabinoids in cancer dissemination was also supported by the in-vitro observations. In particular low OEA concentrations, which were in the range of those observed in human and mouse plasma, promoted cell migration, for which G_i_- or G_q_/11 protein mediated activation of Erk pathways [38] resembling other G_i_- or G_q_/11 activators [39] or a specific effect through OEA-targeted G-protein coupled receptors including GPR55 and GPR119 may be responsible. The inhibition of tumor cell migration at high OEA concentrations, by contrast, possibly owes to the activation of PPARs [40, 41] that are known targets of OEA [3]. In the healthy mouse paw tissue OEA concentrations were in the range of these inhibitory concentrations but concentrations in vitro cannot be directly transferred to the in vivo situation because monoculture conditions do not picture the microenvironment and immune-tumor interactions. Nevertheless, in vitro data support the idea that decreasing endocannabinoid levels favor tumor growth and spreading.

The duality of the OEA effects adds to the controversy about cannabinoid actions on tumor growth. From previous studies it may be inferred that endocannabinoids generally have dual functions in cancer because of their complex effects on tumor cells, endothelial cells and immune cells so that the outcome of endocannabinoid changes likely depends on the local composition of cannabinoid receptors, orphan G-protein coupled receptors and nuclear receptors. For example, GPR55 promotes tumor cell proliferation and angiogenesis [42], CB_1_ inhibits tumor growth and CB_2_ likely promotes tumor-tolerant immune cells. However, opposite effects such as tumor killing by CB_2_ agonists have also been observed [43]. In addition, effects depend on the endocannabinoid concentrations as shown by the in-vitro data. The re-raise of OEA upon metastasis and the correlation of OEA plasma concentrations with the number of metastases may be due to a redistribution or polarization of the immune cells and reflect an attempt to attack the tumor cells. Compared with the in vitro OEA concentrations needed to block migration, plasma OEA levels remained low. Hence, the re-raise of OEA in metastatic cancer may rather indicate frustrate immune stimulation, which was stronger with more metastases but does not indicate successful tumor cell killing.

A further finding of this study was that in addition to OEA, high plasma concentrations of 2-AG were associated with tumor progression and possibly favor it. Previously reported 2-AG values in plasma ranged between 0.4 and 7.5 ng/ml [44], which corresponds to the concentrations measured in the plasma of the present healthy subjects, up to 20 ng/mL [45] in obese subjects. In the present subjects, circulating 1+2-AG concentrations were weakly positively correlated with the patients' body mass index (Spearman's ρ^2^: 0.031, p = 0.001). Therefore, weight loss might be associated with a “readjustment” of individual normal levels but body weight does not explain the high 1+2-AG observed in metastatic cancer, which rather reduces body weight (presently 72 ± 21 kg versus 74 ± 15 kg in patients without metastases).

Considering the present observations of several regulatory consequences on endocannabinoids during cancer, the shift in endocannabinoid plasma profiles might be a candidate biomarker to assess disease progression. In particular, the time courses of endocannabinoid plasma concentrations during tumor cell spreading and establishment of metastases in mice suggested such progression dependency. To test the utility of the human data as a biomarker, endocannabinoid concentrations were submitted to binary logistic regression to identify the cut-off concentration where the probability of metastases was greater than chance but this failed, and on the basis of endocannabinoid concentrations, neither alone nor combined, a prediction of the existence of metastases was not achieved. Thus, circulating endocannabinoid concentrations reflect cancer associated changes but due to the complexity of cannabinoid regulations [19], cross-sectionally assessed endocannabinoid concentrations cannot be exploited as a sensitive biomarker. This does not necessarily extent to longitudinal observations during cancer progression, for which the mouse data provides conceptual support.

The endocannabinoid system was subject to cancer-associated regulations to an extent that led to measurable changes in circulating endocannabinoid levels. In particular, the present analyses showed that various tumors and metastases are associated with reduced circulating ethanolamide endocannabinoids. The changes in circulating endocannaboinds probably do not yet qualify as a biomarker for cancer progression. Nevertheless, given the various major influences on circulating endocannabinoids, the fact that cancer-associated changes were detectable at a statically significant level may reflect the importance of the endocannabinoid system as a factor of cancer progression.

## METHODS

### Animal and molecular study

### Mouse melanoma model

The animal experiments were approved by the regional Ethics Committee for animal research (Darmstadt, Germany) and were in line with the European and German regulations for animal research, in particular the rules of cancer research. Male 8 - 12 weeks-old C57BL/6J mice were used. The mice had free access to food and water, were maintained in climate-controlled rooms at a 12-h light-dark cycle. Mouse melanoma cells (ATCC B16) originating from C57BL/6J mice were grown in 1:1 Dulbecco's modified Eagle medium (DMEM) with 10% supplemental fetal bovine serum and 1% penicillin/streptomycin. The cells were kept in an incubator at 37 °C, 95% humidity and 5% CO_2_ atmosphere, harvested by trypsinization, suspended in cell culture medium and injected subcutaneously into the plantar pad of the left hind paw (1 × 10^4^ cells in 20 μl of culture medium) under 1.5 - 2% isoflurane anesthesia. Cell culture medium without tumor cells was injected in control mice. For induction of metastatic cancer, B16 cells were suspended in 0.9% saline and injected through the tail vein (1 × 10^5^ cells in 50 μl) after taking baseline plasma samples. Eight to nine mice per group were used for plasma samples and 18 mice for paw tissue.

Fourteen days (paw) or three weeks (metastatic) after tumor cell injection mice were finally anaesthetized with isoflurane and blood samples were collected by cardiac puncture in heparinized tubes. After intravenous tumor cell injection additional samples were collected during the course of the disease by puncturing the retro-orbital plexus. After centrifugation, plasma was frozen on dry ice and stored at −80 °C pending analysis. The subcutaneous fat tissue of the paws with the B16 tumor or respective tissue from control mice was dissected, rapidly frozen on dry ice and stored at −80 °C. In metastatic cancer the number and localization of metastases were assessed during necropsy.

### Cancer cell migration assay

To assess the effects of OEA on tumor cell migration a standard scratch assay [20] was employed. B16 mouse melanoma cells (5 × 10^5^ cells) were seeded in 6-well culture plates and grown to sub-confluence (90%). Cultures were then scratched with a blue pipet tip. Just before scratching the culture OEA (0.01, 0.1, 0.5, 1, 2, 10 or 100 μM in 2 μl ethanol) was added to the culture medium (DMEM without FBS) and thoroughly mixed. Control cultures received the respective volume of vehicle. Images of the wound were captured with an inverted Zeiss microscope (Axiovert) at times 0, 6, 24 and 30 h. Images were analyzed using the Autmess modul of Axiovision (Zeiss) in terms of the wound area, relative to the area at 0 h, and in terms of the number of cells within the wound area.

### Human data

Human data was available from an observational cross-sectional investigation of 304 cancer patients (147 men, 157 women, aged 32 - 87 years, Table 1) consecutively recruited from outpatients (n = 286) and inpatients (n = 18) of the Department of Hematology and Oncology, Nordwest Hospital, Frankfurt am Main, Germany. The Declaration of Helsinki on Biomedical Research Involving Human Subjects was adhered to and data and blood collection was approved by the Ethics Committees of the Medical Faculty of the Goethe University and of the Landesärztekammer Hessen, Frankfurt am Main, Germany. Informed written consent from each participating subject had been obtained (Table 1). Tumor staging at diagnosis was recorded with the TNM Classification of Malignant Tumors by the Union for Cancer Control (UICC at http://www.uicc.org/tnm). Venous blood samples were collected to Na+-EDTA tubes and centrifuged at 3,000 rpm for 10 min. Plasma was frozen at −80 °C pending analysis. Aged and sex matched samples for comparative analyses of endocannabinoid concentrations were drawn from a cohort of 300 healthy subjects in total (99 men, 201 women, aged 18 - 57 years), enrolled in the Occupational Health Service ambulance at the University Hospital of Frankfurt am Main, Germany. Enrollment and sample acquisition had been approved by the local Ethics committee, and informed written consent from each participating subject had been obtained.

### Analysis of endocannabinoid concentrations

Concentrations of AEA, PEA, 1- and 2-AG and OEA were quantified by means of liquid chromatography tandem mass spectrometry assays as described elsewhere [21]. Briefly, plasma samples were spiked with the internal standards and liquid-liquid extracted. After evaporation, samples were reconstituted with 50 μL of acetonitrile and injected into the LC-MS/MS system. Separation of the analytes was carried out under gradient conditions using a Luna C18 column. Precursor-to-product ion transitions of *m/z* 346→259 for AEA, *m/z* 354→267 for AEA-d_8_, *m/z* 298→268 for PEA, *m/z* 302→272 for PEA-d_4_, *m/z* 377→303 for 2-AG and 1-AG, *m/z* 382→303 for 2-AG-d_5_ and 1-AG-d_5_, *m/z* 324→86 for OEA, and *m/z* 326→86 for OEA-d_2_ were used for quantitation. Concentrations of the calibration standards, quality controls and unknowns were evaluated by Analyst software (version 1.5; AB Sciex, Darmstadt, Germany). Variations in accuracy and intra-day and inter-day precision (n = 6 for each concentration, respectively) were < 15% over the range of calibration. In an acidic environment, 2-AG undergoes acyl migration converting it to its more stable regio-isomer 1-AG [22] (Figure 1 bottom right). Therefore, statistical analyses were done on the sum of their concentrations. The lower limits of quantification were 0.1 ng/ml for anandamide, 0.25 ng/ml for 1-AG and 2-AG, and 0.5 ng/ ml for PEA and OEA.

### Statistics

Endocannabinoid concentrations were compared between groups or matched subsamples of groups using analyses of variance (ANOVA), t-tests or Mann-Whitney U tests according to the data distribution. Trends were analyzed using the Jonckheere–Terpstra trend test. Further analyses consisted of correlations (Spearman's ρ) and χ^2^ statistics. The wound area from the cell migration assay was analyzed by means of repeated-measures ANOVA, with within-subjects factors “time” and between-subjects factors “OEA concentration”, and for the 24 h observations by means of ANOVA with the between-subjects factor “OEA concentration”. T-tests were used for post-hoc comparisons against vehicle. The α level was set at 0.05 and corrected for multiple testing (Bonferroni). Statistics were done with the SPSS software package (version 21 for Linux, IBM SPSS Inc., Chicago, USA).
